# Endoscopy changing the treatment of congenital nasolacrimal duct obstruction

**DOI:** 10.5935/0004-2749.2024-0087

**Published:** 2024-05-09

**Authors:** Silvana Artioli Schellini, Patricia Mitiko Santello Akaishi

**Affiliations:** 1 Departamento de Anestesiologia e Especialidades Cirúrgicas, Faculdade de Medicina de Botucatu, Universidade Estadual Paulista “Prof. Júlio de Mesquita Filho”, Botucatu, SP, Brazil; 2 Departamento de Oftalmologia e Otorrinolaringologia, Faculdade de Medicina de Ribeirão Preto, Universidade de São Paulo, Ribeirão Preto, SP, Brazil

Dear Editor,

The treatment of congenital nasolacrimal obstruction (CNLDO), which is characterized by tearing and the “messy eye” starting in the 1^st^ or 2^nd^ week of life^(1)^, is changing due to a better understanding of this condition from the use of nasal endoscopy^(2,3)^.

By approximately the 8^th^ month of intrauterine life, the embryonic development of the lacrimal drainage system is completed. However, it may not be fully opened at birth, causing tearing in up to 20% of newborns^(4)^.

Almost all infants with a “membranous” CNLDO can experience spontaneous resolution of the condition during the first few months of life with proper hydrostatic massage. However, a small proportion of children with “complex” obstruction, which is characterized by epiphora associated with secretion and an enlarged lacrimal sac, may require other therapeutic procedures^(1)^.

Regardless of the child’s age, lacrimal sac enlarged and failure of treatment with hydrostatic massage are indications for probing. Under general anesthesia, the Bowman probe is introduced through the lacrimal punctum and pursued through the lacrimal drainage system until it reaches the nasal cavity, with the intention of clearing the obstruction of the nasolacrimal duct ostium. The probe entry into the inferior nasal meatus can be indirectly checked with the “metal-to-metal” touch, lacrimal duct irrigation, or dacryocystography, but none are as efficient as direct probe visualization through nasal endoscopy^(1,2,3)^.

The use of a nasal endoscope to control lacrimal probing in CNLDO is fairly recent as it was only introduced in 1997^(5)^. However, visualizing the opening of the lacrimal drainage system in the inferior nasal meatus is a decisive factor in the treatment of CNLDO as it can differentiate between total or partial obstruction and can determine whether the obstruction is caused by a thin or thick membrane (“membranous” obstruction), stenosis of the ostium, or impacted inferior turbinate. “Complex” obstructions can result from embryological changes, including different degrees of stenosis up to the non-formation of the bone canal of the nasolacrimal duct. These changes make it difficult or even prevent the probe from entering the nasal cavity^(3,4)^.

Endoscopy enables the determination of the location and type of obstruction, directing treatment to the cause of the obstruction. The advantages of observing the passage of the probe through the endoscope are clear and increase the chances of therapeutic success, as it is possible to visually identify and treat the submucosal paths and avoid false passages, resulting in less re-ex posure of the child to general anesthesia to repeat probing or other procedures. Additionally, it facilitates intubation of the tear’s ducts with the silicone thread, leading to a lower risk of iatrogenic events. Situations requiring microsurgical interventions, including removal of thick membranes, submucosal passages, and associated intra-nasal cysts, or ostial stenosis enlargement, can be treated immediately after their discovery. If a “complex” obstruction is diagnosed, and the probe does not reach the nasal cavity, endonasal or transcutaneous dacryocystorhinostomy can be performed^(4)^.

Management decisions should be based on endoscopic probing. The physician must provide all treatment options arising from the diagnosis established by the endoscopic examination, ranging from subluxation of the inferior turbinate to dacryocystorhinostomy, and discuss these with the family before the endoscopic probing. Therefore, endoscopic probing is the most ideal and comprehensive procedure to diagnose and treat CNLDO.
